# Household Contacts of Leprosy Patients in Endemic Areas Display a Specific Innate Immunity Profile

**DOI:** 10.3389/fimmu.2020.01811

**Published:** 2020-08-11

**Authors:** Anouk van Hooij, Maria Tió-Coma, Els M. Verhard, Marufa Khatun, Khorshed Alam, Elisa Tjon Kon Fat, Danielle de Jong, Abu Sufian Chowdhury, Paul Corstjens, Jan Hendrik Richardus, Annemieke Geluk

**Affiliations:** ^1^Department of Infectious Diseases, Leiden University Medical Center, Leiden, Netherlands; ^2^Rural Health Program, The Leprosy Mission International Bangladesh, Dhaka, Bangladesh; ^3^Department Cell and Chemical Biology, Leiden University Medical Center, Leiden, Netherlands; ^4^Department of Public Health, Erasmus MC, University Medical Center Rotterdam, Rotterdam, Netherlands

**Keywords:** innate immunity, lateral flow test, diagnostics, *M. leprae*, UCP-LFA, leprosy

## Abstract

Leprosy is a chronic infectious disease, caused by *Mycobacterium leprae*, that can lead to severe life-long disabilities. The transmission of *M. leprae* is continuously ongoing as witnessed by the stable new case detection rate. The majority of exposed individuals does, however, not develop leprosy and is protected from infection by innate immune mechanisms. In this study the relation between innate immune markers and *M. leprae* infection as well as the occurrence of leprosy was studied in household contacts (HCs) of leprosy patients with high bacillary loads. Serum proteins associated with innate immunity (ApoA1, CCL4, CRP, IL-1Ra, IL-6, IP-10, and S100A12) were determined by lateral flow assays (LFAs) in conjunction with the presence of *M. leprae* DNA in nasal swabs (NS) and/or slit-skin smears (SSS). The HCs displayed ApoA1 and S100A12 levels similar to paucibacillary patients and could be differentiated from endemic controls based on the levels of these markers. In the 31 households included the number (percentage) of HCs that were concomitantly diagnosed with leprosy, or tested positive for *M. leprae* DNA in NS and SSS, was not equally divided. Specifically, households where *M. leprae* infection and leprosy disease was not observed amongst members of the household were characterized by higher S100A12 and lower CCL4 levels in whole blood assays of HCs in response to *M. leprae*. Lateral flow assays provide a convenient diagnostic tool to quantitatively measure markers of the innate immune response and thereby detect individuals which are likely infected with *M. leprae* and at risk of developing disease or transmitting bacteria. Low complexity diagnostic tests measuring innate immunity markers can therefore be applied to help identify who should be targeted for prophylactic treatment.

## Introduction

Leprosy is a debilitating disease that is one of the leading causes of long-term nerve damage worldwide (1). Multidrug therapy (MDT) effectively kills *Mycobacterium leprae*, the causative agent of leprosy, providing an effective cure when treatment is initiated timely (2, 3). To achieve elimination of leprosy, however, it is vital to not only treat adequately and timely but also to prevent transmission (4). The stable new case detection rates in many leprosy endemic countries (5) indicate that MDT insufficiently reduces transmission of *M. leprae*. Recognition of the often subtle cardinal clinical signs is of major importance for leprosy diagnosis (6). The declaration of the WHO in 2000 that leprosy had been eliminated as a public health problem (7), however, caused a reduction of leprosy control activities. The reduced intensity in case detection activities and training in the diagnosis and treatment of leprosy results in many cases that remain undetected for several years (8), allowing the transmission of *M. leprae* to continue.

Contacts close to leprosy patients have a higher risk of acquiring the infection, especially when the patients carry high bacillary loads (9–11). Fortunately, the majority of exposed individuals is naturally immune to *M. leprae* infection (12). Host immunity also determines the clinical phenotype of leprosy, ranging from paucibacillary (PB) patients with a strong proinflammatory response (Th1/Th17) leading to bacterial control to multibacillary (MB) patients with an anti-inflammatory immune response (Th2) producing large quantities of antibodies but unable to control the bacteria (13, 14). In the innate immune response macrophages are critical mediators that define the course of *M. leprae* infection and clinical outcome. In PB patients IL-15 induces antimicrobial activity and the vitamin D-dependent antimicrobial program in macrophages restricting bacterial dissemination (proinflammatory M1 macrophages) (15). In contrast, in MB patients a scavenger receptor program is induced by IL-10, leading to foam cell formation by increased phagocytosis of mycobacteria and oxidized lipids, and persistence of *M. leprae* (anti-inflammatory M2 macrophages) (16, 17).

Markers of the innate immune response can thus be helpful to identify *M. leprae* infected individuals who are prone to develop leprosy disease and thereby, since they are unable to kill and remove *M. leprae*, contribute to the ongoing transmission. No practical tools are yet available to identify individuals that should be prioritized for prophylactic treatment. Recently, biomarkers for leprosy and *M. leprae* infection were identified (18, 19), including serum proteins that play a role in innate immunity. For example, S100A12 is required to decrease *M. leprae* viability in infected macrophages (20). CCL4 and IP-10 attract innate immune cells such as natural killer (NK) cells and monocytes, whereas IL-1Ra-stimulated monocytes turn into M2 macrophages that produce high levels of the anti-inflammatory cytokine IL-10 (21).

Two other identified biomarkers (19) that play a role in the innate immune system were contrasting acute phase proteins: anti-inflammatory ApoA1 and pro-inflammatory CRP. ApoA1 inhibits the recruitment of monocytes and macrophage chemotaxis (22), whereas CRP can recognize pathogens and activate the classical complement pathway (23). Together with αPGL-I IgM, the well-established biomarker for MB leprosy (24), the identified biomarkers were implemented in quantitative up-converting phosphor lateral flow assays (UCP-LFAs) (19). These user-friendly tests are applicable in resource-limited settings, essential for diagnostic tools in large-scale contact screening of leprosy contacts, and provide quantitative results. The latter allows monitoring of drug treatment as well as discriminating high from low responders.

Previously, we analyzed nasal swabs (NS) and slit-skin smears (SSS) of household contacts (HCs) of MB leprosy patients with high bacillary loads for the presence of *M. leprae* DNA (25). Here we analyzed the same individuals to examine the correlation of the presence of *M. leprae* DNA with the levels of innate immune markers. *M. leprae* DNA in NS indicates colonization of the HC with the bacterium, but not invasion of the tissue. Detection of *M. leprae* DNA in SSS does indicate that a HC is infected. In this study, levels of ApoA1, CCL4, CRP, IL-1Ra, IL-6, IP-10, αPGL-I IgM, and S100A12 were determined by UCP-LFAs in supernatants of 24 h *M. leprae* antigen-stimulated whole blood assays (WBA) addressing newly diagnosed MB patients with a high bacteriological index (BI) and their HCs in Bangladesh.

## Materials and Methods

### Study Participants

The cohort used in this study originates from four districts in Bangladesh (Nilphamari, Rangpur, Panchagar, and Thakurgaon) and has been extensively described previously (25). The prevalence of leprosy in these districts was 0.9 per 10,000 and the new case detection rate 1.18 per 10,000 (Rural health program, the leprosy mission Bangladesh, yearly district activity report 2018).

Between July 2017 and May 2018, newly diagnosed leprosy patients (index case; *n* = 31) with BI ≥2 and between 3 and 15 HCs per index case (*n* = 279) were recruited (25). Leprosy was diagnosed based on clinical and bacteriological observations and classified as MB or PB as described by the WHO (5) and the BI was determined. HCs were examined as well for signs and symptoms of leprosy upon recruitment and followed up yearly for surveillance of new case occurrence for ≥24 months after sample collection.

Control individuals without known contact to leprosy or TB patients and without clinical disease symptoms from the same leprosy endemic area (EC) were included and assessed for the absence of clinical signs and symptoms of leprosy and TB. Staff of leprosy or TB clinics were excluded as EC.

### Household Contacts

The coding system used to describe physical and genetic distance of contacts from the patient has been extensively described previously (26). In short, four categories of physical distance are relevant for this study:

- KR: contacts living under the same roof and the same kitchen
- K: contacts living under a separate roof but using the same kitchen
- R: contacts living under the same roof, not using the same kitchen
- N1: next-door neighbors

In this study the KR and R group were considered as one group.

For genetic distance seven categories were defined: spouse (M), child (C), parent (P), sibling (B), other relative (O), relative in-law (CL, PL, BL, or OL), and not family related (N). CL, PL, and OL were considered as one group in this study, referred to by OL.

### Ethics

This study was performed according to the Helsinki Declaration (version Fortaleza, Brazil, October 2013). The studies involving human participants were reviewed and approved by the Bangladesh Medical Research Council/National Research Ethics Committee (BMRC/NREC/2010-2013/1534). Participants were informed about the study-objectives, the samples and their right to refuse to take part or withdraw from the study without consequences for their treatment. Written informed consent was obtained before enrolment. All patients received treatment according to national guidelines.

### Sample Collection

SSS from the earlobe and NS were collected for detection of *M. leprae* DNA as described previously (25). For the WBA, 4 ml venous blood was drawn and 1 ml was applied directly to a microtube precoated with 10 μg *M. leprae* whole cell sonicate (WCS) or without stimulus (Med). After 24 h incubation at 37°C the microtube was frozen at −20°C, shipped to the LUMC and stored at −80°C until further analysis.

### DNA Isolation and RLEP PCR/qPCR

DNA isolated from the NS and SSS was used to perform RLEP PCR and qPCR as described previously (25). Presence of *M. leprae* DNA was considered if a sample was positive for RLEP qPCR with a Ct lower than 37.5 or was positive for RLEP PCR at least in two out of three independently performed PCRs to avoid false positives.

### UCP-LFAs

Levels of αPGL-I IgM, CRP, IP-10, S100A12, ApoA1, IL-6, IL-1Ra, and CCL4 in WBA supernatant were analyzed using UCP-LFAs. αPGL-I IgM, CRP, IP-10, S100A12, and ApoA1 UCP-LFAs have been described previously (18, 19). IL-6, IL-1Ra, and CCL4 UCP-LFAs were produced similarly, with a Test line of 200 ng MQ2-39C3 (IL-6; BioLegend, San Diego, USA), AF280 (IL-1Ra), and clone 24006 (CCL4) (R&D systems, Minneapolis, USA) and a Flow Control line with 100 ng Goat-anti-Rat (IL-6; R5130, Sigma-Aldrich), Goat-anti-Mouse (IL-1Ra; M8642; Sigma-Aldrich), and Rabbit-anti-Goat (CCL4; G4018, Sigma-Aldrich). Complementary antibodies were conjugated to the UCP particles, MQ2-13A5 (BioLegend, San Diego, USA), clone 10309 (IL-1Ra), and AF-271-NA (CCL4) (R&D systems, Minneapolis, USA). Yttrium fluoride upconverting nano materials (200 nm, NaYF4:Yb3+,Er 3+) functionalized with polyacrylic acid were obtained from Intelligent Material Solutions Inc. (Princeton, New Jersey, USA).

To perform the UCP-LFAs WBA supernatant was diluted 5-fold (IP-10, IL-1Ra and CCL4), 50-fold (IL-6, αPGL-I IgM and S100A12), 500-fold (CRP) and 5,000-fold (ApoA1) in high salt buffer (100 mM Tris pH 8, 270 mM NaCl, 1% (w/v) BSA, 1% (v/v) Triton X-100). As WCS stimulation does not affect the levels of ApoA1, CRP, and αPGL-I IgM these three markers were only determined in medium. Strips were analyzed using a UCP dedicated benchtop reader (UPCON; Labrox, Finland). Results are displayed as the ratio value between Test and Flow-Control signal based on relative fluorescence units (RFUs; excitation at 980 nm and emission at 550 nm) measured at the respective lines.

### Statistical Analysis

GraphPad Prism version 8.1.1 for Windows (GraphPad Software, San Diego CA, USA) was used to perform Mann-Whitney *U*-tests, Kruskal-Wallis with Dunn's correction for multiple testing, Wilcoxon matched-pairs signed rank test, plot receiver operating characteristic (ROC) curves, and calculate the area under curve (AUC). The Pearson correlation coefficient and the corresponding *p*-values and heatmap were also determined using GraphPad Prism.

## Results

### *M. leprae* DNA in Nasal Swabs/Slit-Skin Smears and the Occurrence of Leprosy in HCs

**Figure 1:**
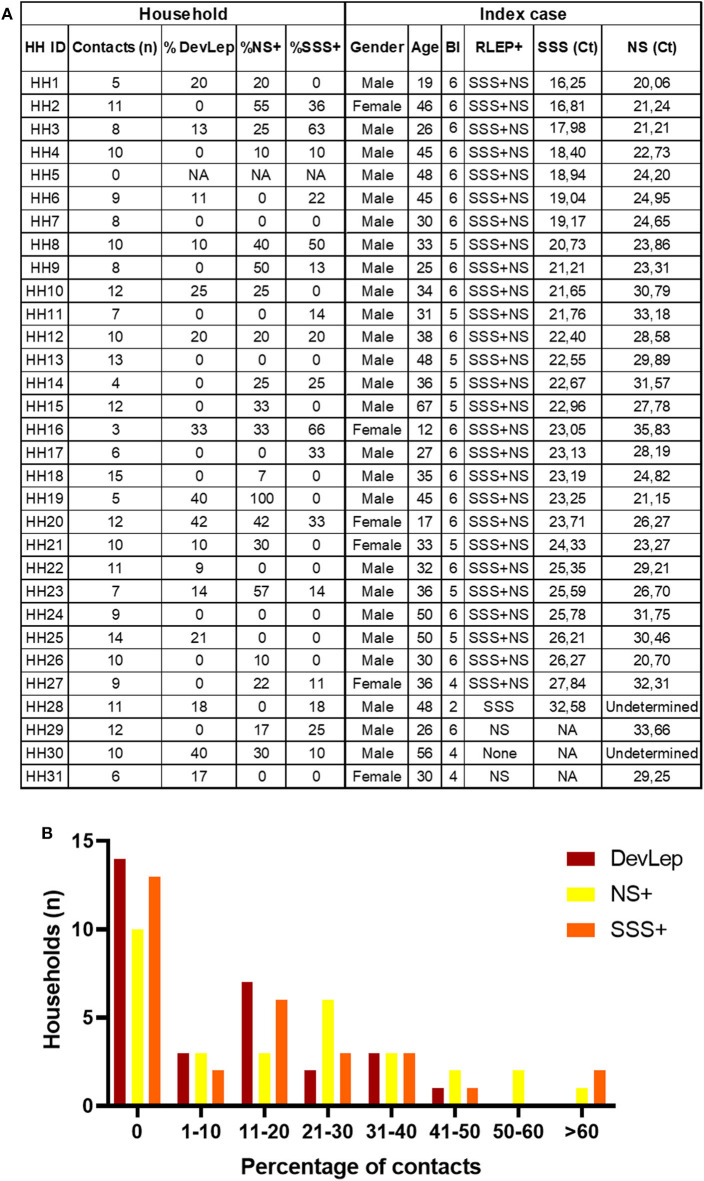
Percentage of *M. leprae* DNA positive nasal swabs/slit-skin smears and occurrence of leprosy in contacts per household. **(A)** Table indicates the number of household contacts per index case, the percentage of contacts that were diagnosed with leprosy during contact screening (%DevLep) and the percentage of contacts with *M. leprae* DNA detected in nasal swabs (%NS+) and slit-skin smears (%SSS+). The characteristics of the index case of each household (HH) are also indicated in this table. RLEP+ indicates whether *M. leprae* DNA was detected in the NS or SSS of the index case, the corresponding Ct values are indicative of the amount of *M. leprae* bacilli in NS and SSS. A low Ct value corresponds to high amounts of bacteria. BI, bacteriological index; NA, Not applicable. **(B)** On the *x*-axis the percentage range of household contacts (HCs) diagnosed with leprosy during contact screening (DevLep; dark red bars), that were *M. leprae* DNA positive in nasal swabs (NS+; yellow bars) or slit-skin smears (SSS+; orange bars) is indicated. The *y*-axis depicts the number of households for the percentage range indicated on the *x*-axis. The number of households within each percentage range was determined using the data table from **(A)**.

The presence of *M. leprae* DNA in NS and SSS of HCs was assessed in 31 households of MB index cases with BI ≥2 (25) (Figure 1). Out of 279 HCs, 29 were diagnosed with leprosy upon first physical investigation at intake, and four were diagnosed with PB leprosy during follow-up. Of the patients diagnosed at intake the majority (93%) had a low bacillary load: 22 were PB and seven were MB, of whom five with BI 0 (MB/BT) and two with BI ≥4 (Supplementary Figure 1). The HCs diagnosed with leprosy at intake (DevLep) were not evenly distributed over the different households: in 14 households none of the HCs had developed leprosy, whereas in the other 17 households, 9–42% suffered from leprosy (Figure 1). Applying previous results on the presence of *M. leprae* DNA (25), indicated that in 10 households *M. leprae* DNA was not detected in any of the HCs in NS and in 13 households all HCs were negative in the SSS. Of the households where *M. leprae* DNA was detected, percentages of colonization varied from 7 to 100% (NS) and for infection from 10 to 66% (SSS; Figure 1). The proportion of *M. leprae* DNA presence in NS or SSS and identified leprosy in HCs upon first physical screening thus varies between households even if the index cases have similarly high bacillary loads.

### ApoA1 and S100A12 Levels Differentiate HCs From EC

**Figure 2:**
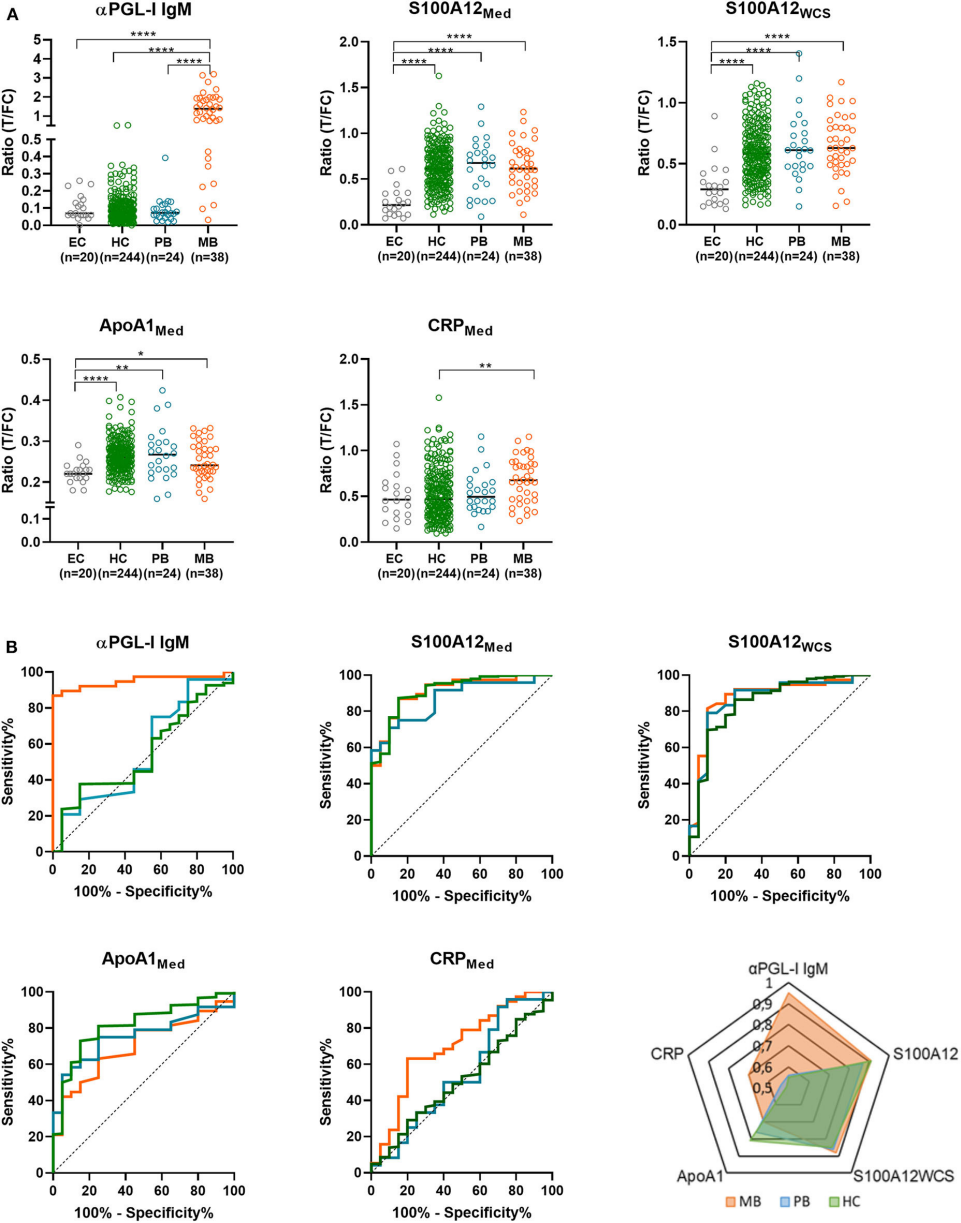
Differentiation of leprosy patients and household contacts (HC) from endemic controls (EC) by immune markers. Whole blood without stimulus (Med) or stimulated with *M. leprae* whole cell sonicate (WCS) was frozen after 24 h. Levels of 8 proteins (αPGL-I IgM, S100A12, ApoA1, CCL4, IP-10, IL-6, IL-1Ra, and CRP) were assessed by up-converting phosphor lateral flow assays (UCP-LFAs) in these whole blood assay supernatants for 31 households of index cases with multibacillary (MB) leprosy (bacteriological index ≥2). **(A)** UCP-LFA ratio values were calculated by dividing the peak area of the test line (T) by the peak area of the flow control line (FC; *y*-axis). As ratio values are marker dependent the *y-*axis scale differs per marker. The levels of MB (orange circles) and paucibacillary (PB; blue circles) patients, household contacts (HC; green circles) and endemic controls (EC; gray circles) were compared using the Kruskal-Wallis test with Dunn's correction for multiple testing. The data of CCL4, IP-10, IL-6, and IL-1Ra were not shown as no significant differences were observed in the levels of these proteins between groups. *P*-values: *p ≤ 0.05, **p ≤ 0.01, ****p ≤ 0.0001. **(B)** Receiver operating characteristic (ROC) curves were computed comparing the levels of αPGL-I IgM, CRP, S100A12, ApoA1 in multibacillary (MB) /paucibacillary (PB) patients and HC to EC. These levels were determined by up-converting phosphor lateral flow assays in supernatant of 24 h *M. leprae* antigen-stimulated whole blood assays (WBA; medium = Med, *M. leprae* whole cell sonicate = WCS). A summary of the areas under the curve (AUC) for MB (orange), PB (blue) and HC (green) is depicted in the spider plot showing the markers in which significant differences were observed (lower right panel).

Levels of αPGL-I IgM, CRP, IP-10, S100A12, ApoA1, IL-6, IL-1Ra, and CCL4 were determined by UCP-LFA in WBA supernatant. Levels of these eight markers in patients (*n* = 62; 38 MB and 24 PB), HCs (*n* = 244) and EC (*n* = 20) without known contact to leprosy patients were compared. Stimulation with *M. leprae* WCS had a significant impact on the CCL4, IL-1Ra, and IL-6 levels (Supplementary Figure 2). Significant differences between the groups were observed for αPGL-I IgM, S100A12_Med_, S100A12_WCS_, ApoA1, and CRP (Figure 2A). Compared to EC, the AUC values for αPGL-I IgM and CRP were significant only for MB patients, whereas ApoA1 and S100A12 levels significantly differed in both MB and PB patients. In HCs, however, the levels of S100A12 were comparable to those in (MB and PB) patients with similar AUCs (ranging from 0.85 to 0.91; Figure 2B). Interestingly, the difference in ApoA1 levels between EC was more profound for HC (AUC:0.81; *p* < 0.0001) than for PB (AUC:0.76; *p* = 0.0039) or MB patients (AUC: 0.7; *p* = 0.0126). As described for other cohorts previously (18), MB patients can be discriminated from HCs based on αPGL-I IgM (*p* < 0.0001) and CRP (*p* = 0.0024), but these markers cannot differentiate PB patients from HCs with similar rates of *M. leprae* DNA presence in NS and SSS (25). These data thus indicate that PB patients and HCs respond similarly to *M. leprae*.

### S100A12 and CCL4 Response Is Associated With the Occurrence of Leprosy in Households

**Figure 3:**
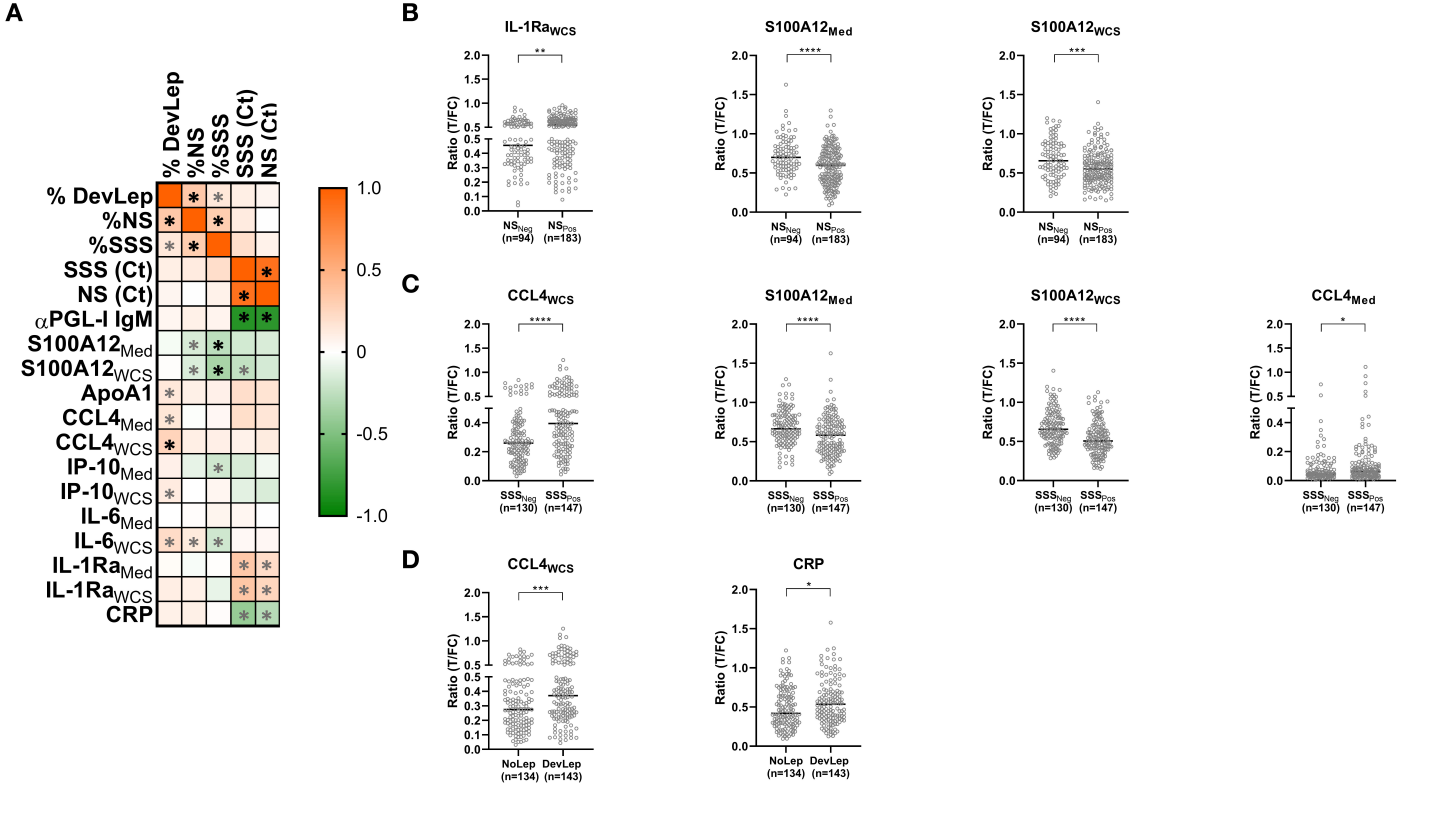
Correlation of leprosy disease and M. leprae infection/colonization status in households with innate immune markers. **(A)** Whole blood without stimulus (Med) or stimulated with *M. leprae* whole cell sonicate (WCS) was frozen after 24 h. Levels of 8 proteins (αPGL-I IgM, S100A12, ApoA1, CCL4, IP-10, IL-6, IL-1Ra, and CRP) were assessed by up-converting phosphor lateral flow assays (UCP-LFAs) in supernatants of WBA for 31 households of index cases with multibacillary (MB) leprosy (bacterial index ≥2). The proportion of household contacts (HCs) diagnosed with leprosy upon first clinical examination (%DevLep) or with *M. leprae* DNA presence in nasal swabs (%NS) or slit-skin smears (%SSS) was calculated per household. These percentages and the RLEP Ct values determined by qPCR in NS and SSS were correlated with the levels of the assessed immune markers. The heatmap indicates the correlation coefficient (R), ranging from −1 (green) to 1 (orange) as determined using GraphPad Prism. Significant correlations (*p* < 0.05) are indicated with an asterisk (*), highly significant (*p* < 0.0001) are indicated with a black asterisk (*). **(B)** Significantly different (*p* < 0.05) levels of immune markers observed in HCs of *M. leprae* DNA positive (NS_Pos_) and negative (NS_Neg_) households. Ratio values (*y*-axis) represent the level of the assessed marker and were determined by dividing the signal of the test line (T) by the signal of the flow control (FC) line of the up-converting phosphor lateral flow assays. **(C)** Significantly different (*p* < 0.05) levels of immune markers observed in HCs of *M. leprae* DNA positive (SSS_Pos_) and negative (SSS_Neg_) households. **(D)** Significantly different (*p* < 0.05) levels of immune markers between HCs living in households where leprosy was diagnosed among contacts (DevLep) and in households where leprosy was not observed (NoLep).

The relationship between disease and infection/colonization status in households was examined into more detail by determining the correlation between the immune markers and the percentage of HCs with detectable *M. leprae* DNA in NS (%NS) and SSS (%SSS) or diagnosed with leprosy (%DevLep) (Figure 3A). A highly significant (*p* < 0.0001) positive correlation was identified for the %DevLep with CCL4_WCS_ and a negative correlation for %SSS with S100A12_Med_ and S100A12_WCS_ (Supplementary Table 1). For a subset of individuals qPCR Ct values were available indicative of the quantity of *M. leprae* DNA in NS (*n* = 105) or SSS (*n* = 71). These Ct values showed an inverse correlation with αPGL-I IgM antibodies in this cohort, indicating a strong positive correlation between the amount of *M. leprae* and the PGL-I antibody titer (25). For IL-1Ra_Med_/IL-1Ra_WCS_ and inversely for CRP, a significant correlation was observed with the Ct values for both NS and SSS as well (Supplementary Table 1).

A cross-sectional analysis was performed to compare households in which HCs developed leprosy to households where this was not observed. The same analysis was performed for households where *M. leprae* DNA was present in NS or SSS of HCs. In households where *M. leprae* DNA was detected in NS significantly lower levels of S100A12_Med_ (*p* < 0.0001) and S100A12_WCS_ (*p* = 0.0005) and higher levels of IL-1Ra_WCS_ were observed (Figure 3B). S100A12 levels were also significantly lower in households where *M. leprae* DNA was detected in SSS (Figure 3C; *p* < 0.0001). CCL4 levels were higher in these households, especially in response to *M. leprae* WCS (*p* < 0.0001). Higher levels of CCL4_WCS_ were also observed in the households where HCs of the primary index case were diagnosed with leprosy upon first physical investigation at intake (*p* = 0.0002) as well as increased levels of CRP (*p* = 0.025; Figure 3D).

The levels of CCL4 and S100A12 showed a significant result in both the correlation and cross-sectional analysis, indicating an association of these markers with leprosy and/or *M. leprae* infection among HCs.

### *M. leprae* Colonization in HCs Correlates With Physical Distance to the Index Case

To examine the influence of the characteristics of the index case (all MB patients with high bacillary loads) on the development of leprosy and *M. leprae* colonization (NS) or infection (SSS) in HCs, a correlation and cross-sectional analysis was performed (Supplementary Figure 3). Cross-sectionally, higher S100A12_Med_ levels were observed in index cases without detectable *M. leprae* DNA in NS of their HCs (*p* = 0.035). No other significant differences were observed in index cases for the other markers nor in the amount of bacteria in SSS or NS. Thus, characteristics of the index case in this cohort have little influence on the observed differences between the households (Figure 1).

**Figure 4:**
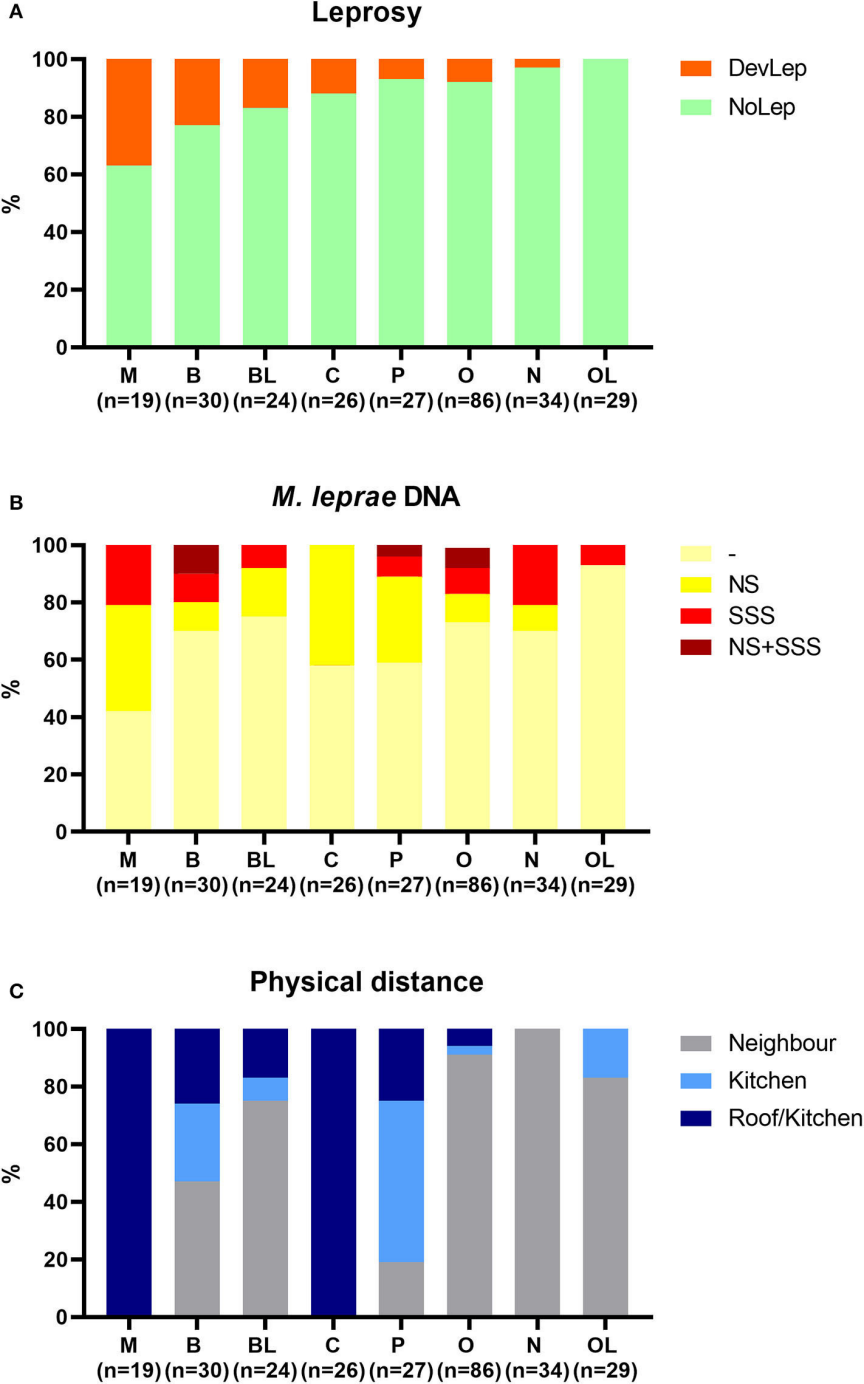
Stratification of household contacts by genetic distance to the index case. Eight different groups were classified for genetic distance: spouse (M), child (C), parent (P), sibling (B), other relative (O), brother/sister in law (BL), other relatives in law (OL), and not family related (N). **(A)** Percentage of individuals diagnosed with leprosy upon first clinical examination (DevLep; orange) stratified by genetic distance and ranked by percentage. **(B)** Percentage of *M. leprae* DNA presence in nasal swabs (NS; yellow), slit-skin smears (SSS; red) or both (NS + SSS; dark red) stratified by genetic distance. **(C)** Distribution of physical distance (Roof/kitchen = dark blue, kitchen = blue, Neighbor = gray) to the index case stratified by genetic distance.

The influence of genetic relationship and physical distance of HCs to the index case was also examined. HCs were stratified by genetic distance against the percentage of leprosy and *M. leprae* DNA presence in NS and SSS in these groups (Figure 4). Development of leprosy was most frequently observed in spouses (37%), followed by siblings (23%) and siblings in law (17%) (Figure 4A). Spouses also showed the highest frequency of *M. leprae* presence in NS and/or SSS (58%), followed by children (42%), and parents (41%) (Figure 4B). Spouses, children, and parents live in the closest proximity of patients (Figure 4C; living under the same roof or sharing a kitchen) and thus have the highest level of exposure. Physical distance indeed correlated significantly (*p* = 0.003; *R*^2^ = 0.8) with the %NS_Pos_ (colonization), though this was not observed for the development of leprosy in HCs (*p* = 0.07; *R*^2^ = 0.44).

**Figure 5:**
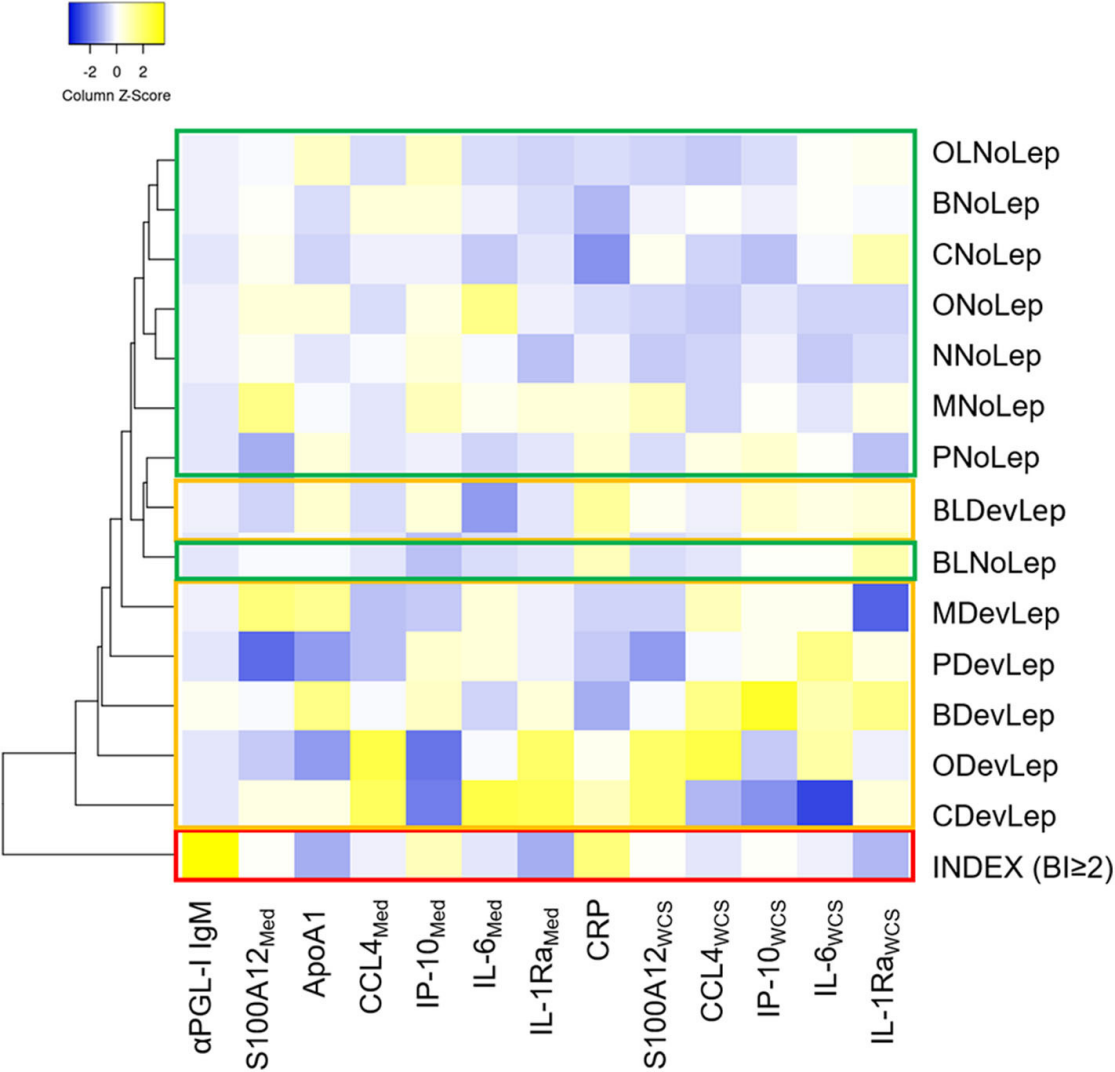
Contacts diagnosed with leprosy upon first clinical screening cluster together based on their immune response, irrespective of genetic distance. Whole blood without stimulus (=Med) or stimulated with *M. leprae* whole cell sonicate (=WCS) was frozen after 24 h. Levels of 8 proteins (αPGL-I IgM, S100A12, ApoA1, CCL4, IP-10, IL-6, IL-1Ra, and CRP) were assessed by up-converting phosphor lateral flow assays in supernatants of whole blood assays (WBA) for 31 households of index cases with multibacillary (MB) leprosy (bacteriological index ≥2). The heatmap shows clustering based on average linkage performed by heatmapper (27) of the median level of eight serum protein markers in contacts diagnosed with leprosy upon first clinical screening of the HCs (DevLep) and without leprosy (NoLep) stratified by genetic distance; spouse (M), child (C); parent (P); sibling (B); other relative (O); brother/sister in law (BL); other relatives in law (OL) and not family related (N). The z-score indicates the deviation from the average level of the marker across groups, higher Z-scores are indicated in yellow and lower Z-scores in blue. Red = index case, yellow = contacts diagnosed with leprosy; green = household contacts without leprosy.

The levels of the innate immune markers were also stratified by genetic distance. Based on the median levels of the assessed markers, the HC groups that were diagnosed with leprosy clustered apart from the HC groups that did not show symptoms of disease (Figure 5). Across the groups with different genetic distance to the index case, similar innate immune mechanisms seem to play a role in the development of leprosy in HCs. Additionally, the index case group clustered apart from all HC groups rendering the assessed markers useful for leprosy diagnostics.

## Discussion

To examine the link between innate immunity and *M. leprae* colonization/infection in HCs, immune markers were assessed in 24 h *M. leprae* antigen-stimulated WBAs by UCP-LFAs. Even though all HCs were exposed to comparable levels of *M. leprae*, as all 31 index cases were MB patients with BI ≥2, there was a difference in the percentage of *M. leprae* DNA presence in NS/SSS and the occurrence of leprosy cases between households. Characteristics of the index case, such as the amount of *M. leprae* bacilli in NS or the αPGL-I antibody titer, had little influence on the development of leprosy nor on *M. leprae* colonization/infection in other household members. Physical distance of HCs to the index case was, however, significantly correlated with *M. leprae* colonization, though not with *M. leprae* infection or development of leprosy demonstrating the role of innate immune responses to remove bacteria.

In this study, S100A12 was associated with a protective response to *M. leprae* colonization/infection in HCs. As previously demonstrated (19), S100A12 also remained a useful marker to discriminate leprosy patients from EC. S100A12 has a dual role inducing both proinflammatory and antimicrobial effects by interacting with different receptors, such as RAGE and TLR4 (28). RAGE expression is associated with disease severity and levels of proinflammatory cytokines in active tuberculosis (TB) (29). Contrary, RAGE is protective against the development of pulmonary TB in mouse models (30) in line with reduction of antimicrobial activity in human macrophages upon TLR2/1 ligand activation by S100A12 knockdown (20). S100A12 thus seems to protect exposed individuals from *M. leprae* colonization and infection, but once infected, S100A12 can contribute to maintain a detrimental, pro-inflammatory state in leprosy patients.

ApoA1 levels in HCs were similar to those in PB patients, suggesting that ApoA1 plays a role in limiting bacterial growth. This is in line with the finding that PB patients showed a similar low rate of *M. leprae* DNA presence in NS and SSS as HCs (25). Increased levels of ApoA1 have been observed in cells exposed to activated complement, where ApoA1 inhibits the formation of the membrane attack complex thereby contributing to complement clearance (31). Decreased levels are associated with destructive chronic inflammation, as ApoA1 exerts anti-inflammatory effects (32). The effects of ApoA1 do, however, not only rely on the protein level but also on the functionality, oxidative modification can for instance transform ApoA1 to an inflammatory agent (33). The role and functionality of ApoA1 in leprosy thus remains to be further elucidated. The influence of ApoA1 on lipid metabolism is of interest as dysfunctional high-density lipoprotein (involved in cholesterol transport to the liver of which the main protein is ApoA1) related to altered ApoA1 levels has been observed in MB patients (34). Moreover, it was suggested that *M. leprae* can directly affect ApoA1 biosynthesis.

Other markers in this study were associated with *M. leprae* colonization (IL-1Ra), whereas CCL4 was associated with infection and disease. These responses were most profound upon stimulation with *M. leprae* WCS, reflecting the innate immune response of these individuals to mycobacterial antigens. Interestingly, in whole blood of BCG-vaccinated infants the production of IL-1Ra and CCL4 was decreased upon stimulation of several TLRs (35). This observed response can be a result of BCG-induced trained innate immunity, which is immunological memory of the innate immune response that leads to an enhanced response to a subsequent trigger (36). Moreover, in Systemic Lupus Erythematosus (SLE) a pathogenic three-marker signature, including high levels of IL-1Ra and CCL4, was identified in monocytes (37). The signature was associated with the immune dysregulation in this autoimmune disease, in which flares occur similar to leprosy reactions (38). High levels of IL-1Ra and CCL4 thus seem indicative of pathogenic innate immune responses, corroborating earlier results on the identification of IL-1Ra and CCL4 as biomarkers associated with a pathogenic immune response to *M. leprae* (18, 19, 39).

One of the challenges of application of host immune markers for diagnostics is the influence of co-morbidities or co-infections on biomarker levels. Helminth infections dampen the Th1 response and increase the risk for MB leprosy (40, 41). A biomarker study to examine the influence of helminth co-infection in leprosy patients is currently ongoing. Moreover, the influence on biomarker levels of co-morbidities, such as diabetes mellitus which is known to increase the risk of active TB (42), on the disease outcome should be further studied. Another issue impeding straightforward implementation of biomarkers is that inflammatory markers are not disease-specific. For example, S100A12 has been described as biomarker for rheumatoid arthritis (43), TB (44) as well as inflammatory bowel disease (45). As the UCP-LFA allows quantitative measurement of biomarkers it would be interesting to compare disease-specific S100A12 levels for these conditions. Taking into account the multiple factors that influence host immune responses, a biomarker signature that combines several innate immune markers is required to identify individuals at risk of developing leprosy. This signature should also be evaluated in other inflammatory conditions.

In conclusion: Frequent exposure of HCs to *M. leprae* results in a continuously active innate immune response. This allows differentiation of HCs from EC by user-friendly diagnostic tests measuring specific serum protein levels. If the innate immune response is sufficient, pathogens, and pathogen-infected cells are being successfully removed. However, prolonged (intense) activation can lead to an immune response directed against the host (46). The resemblance of the innate immune response of PB patients and HCs observed in this and previous studies (19, 39) indicates that PB leprosy can be a result of an imbalance in innate immunity. HCs that do not develop disease seem to effectively clear the bacteria without overactivation of the innate immune response. Elucidation of this delicate balance in innate immune responses by quantitation of appropriate biomarker signatures (47) can contribute to the identification of individuals at risk of developing leprosy upon *M. leprae* exposure. To gain more insight in this balance longitudinal analysis is required, which is currently ongoing. Diagnostic user-friendly rapid tests, as applied in this study, that allow quantitative measurement of combinations of innate immune markers represent useful tools to identify individuals that could benefit from prophylactic treatment.

## Data Availability Statement

The raw data supporting the conclusions of this article will be made available by the authors, without undue reservation.

## Ethics Statement

The studies involving human participants were reviewed and approved by the local ethical board in Bangladesh (BMRC/NREC/2010-2013/1534). Written informed consent to participate in this study was provided by the participants' legal guardian/next of kin.

## Author Contributions

AG, AH, and JR: designed research. AS, KA, and MK: enrolled patients, performed, and registered clinical diagnosis. AH, MT-C, EV, DJ, ET, and MK: performed experiments. PC and KA: resources. AH, MT-C, and AG: analyzed the data. AH and AG: wrote the paper. All authors: critically reviewed and agreed with the manuscript.

## Conflict of Interest

The authors declare that the research was conducted in the absence of any commercial or financial relationships that could be construed as a potential conflict of interest. The reviewer RP declared a past co-authorship with the authors MT-C and AG to the handling Editor.
