# Supplementary material for: Household Contacts of Leprosy Patients in Endemic Areas Display a Specific Innate Immunity Profile
Source: Front Immunol. 2020 Aug 11;11:1811. doi: 10.3389/fimmu.2020.01811 (PMC7431626; doi:10.3389/fimmu.2020.01811)
Supplement: Supplementary file 1 [file Data_Sheet_1.pdf]

Supplementary material

Household contacts of leprosy patients in endemic areas display a specific innate immunity profile

Anouk van Hooij[1,*], Maria Tió-Coma[1,*], Els M. Verhard[1], Marufa Khatun[2], Khorshed Alam[2], Elisa Tjon Kon Fat[4], Danielle de Jong[4], Abu Sufian Chowdhury[2], Paul Corstjens[4], Jan Hendrik Richardus[3], and Annemieke Geluk[1]

[1]Dept. of Infectious Diseases, Leiden University Medical Center, The Netherlands; [2]Rural Health Program, The Leprosy Mission International Bangladesh, Nilphamari, Bangladesh; [3]Dept. of Public Health, Erasmus MC, University Medical Center Rotterdam, The Netherlands. [4]Dept. Cell and Chemical Biology, Leiden University Medical Center, The Netherlands.

* these authors contributed equally

Correspondence to:

Prof. dr. A. Geluk, Dept. of Infectious Diseases, LUMC

PO Box 9600, 2300 RC Leiden, The Netherlands.

Tel: +31-71-526-1974 ; Fax +31-71-526-5267; E-mail: a.geluk@lumc.nl

Supplementary Table S1 ***Correlation of leprosy disease and M. leprae infection/colonization status in households with innate immune markers.***

	P-value					Correlation Coefficient (R)				
	% DevLep	%NS	%SSS	SSS (Ct)	NS (Ct)	% DevLep	%NS	%SSS	SSS (Ct)	NS (Ct)
% DevLep		1,83E-09	0,01	0,48	0,62	1,000	0,333	0,146	0,085	0,050
%NS	1,83E-09		4,56E-08	0,30	0,93	0,333	1,000	0,304	0,125	-0,009
%SSS	0,01	4,56E-08		0,10	0,44	0,146	0,304	1,000	0,201	0,076
SSS (Ct)	0,48	0,30	0,10		2,87E-17	0,085	0,125	0,201	1,000	0,890
NS (Ct)	0,62	0,93	0,44	2,87E-17		0,050	-0,009	0,076	0,890	1,000
αPGL-I IgM	0,33	0,12	0,26	2,32E-21	2,64E-28	0,055	0,089	0,064	-0,852	-0,831
$S100A12_{Med}$	0,46	0,005	2,53E-06	0,14	0,12	-0,042	-0,159	-0,264	-0,178	-0,154
$S100A12_{WCS}$	0,83	0,01	5,49E-10	0,04	0,08	0,012	-0,146	-0,343	-0,242	-0,171
ApoA1	0,01	0,10	0,13	0,10	0,08	0,144	0,093	0,085	0,197	0,171
$CCL4_{Med}$	0,01	0,81	0,41	0,09	0,11	0,148	-0,014	0,047	0,205	0,156
$CCL4_{WCS}$	1,35E-06	0,08	0,09	0,52	0,23	0,270	0,098	0,096	0,078	0,118
$IP\text{-}10_{Med}$	0,16	0,08	0,001	0,19	0,53	0,081	-0,100	-0,189	-0,158	-0,063
$IP\text{-}10_{WCS}$	0,04	0,92	0,34	0,34	0,12	0,119	-0,006	0,054	-0,115	-0,153
$IL\text{-}6_{Med}$	0,91	0,81	0,30	0,67	0,99	-0,007	0,014	0,060	0,051	0,001
$IL\text{-}6_{WCS}$	0,0001	0,02	0,001	0,73	0,65	0,215	0,128	-0,193	0,042	0,045
$IL\text{-}1Ra_{Med}$	0,66	0,62	0,94	0,004	0,03	0,025	-0,029	0,004	0,336	0,215
$IL\text{-}1Ra_{WCS}$	0,07	0,12	0,10	0,003	0,01	0,103	0,089	-0,094	0,351	0,252
CRP	0,12	0,11	0,81	0,0004	0,003	0,089	0,092	0,014	-0,409	-0,287

Whole blood without stimulus (Med) or stimulated with *M. leprae* whole cell sonicate (WCS) was frozen after 24 hours. For 31 households of index cases with multibacillary leprosy (bacteriolgocial index ≥ 2), levels of 8 proteins (αPGL-I IgM, S100A12, ApoA1, CCL4, IP-10, IL-6, IL-1Ra and CRP) were assessed by up-converting phosphor lateral flow assays (UCP-LFAs) in whole blood assay supernatants. Per household the percentage of household contacts (HCs) diagnosed with leprosy upon first clinical screening (%DevLep) or positive for *M. leprae* DNA in nasal swabs (%NS) or skin slit smears (%SSS) at that same time, was calculated. Correlation between these percentages and the RLEP Ct values, determined by qPCR in NS and SSS (Tio-Coma et al., 2020), with the levels of the assessed immune markers was determined. The p-value and the corresponding correlation coefficient (R) are shown. Significant p-values (green) indicate which innate immune markers are correlated with the amount of bacteria in NS and SSS assessed by qPCR or are correlated with the %DevLep, %NS or %SSS.

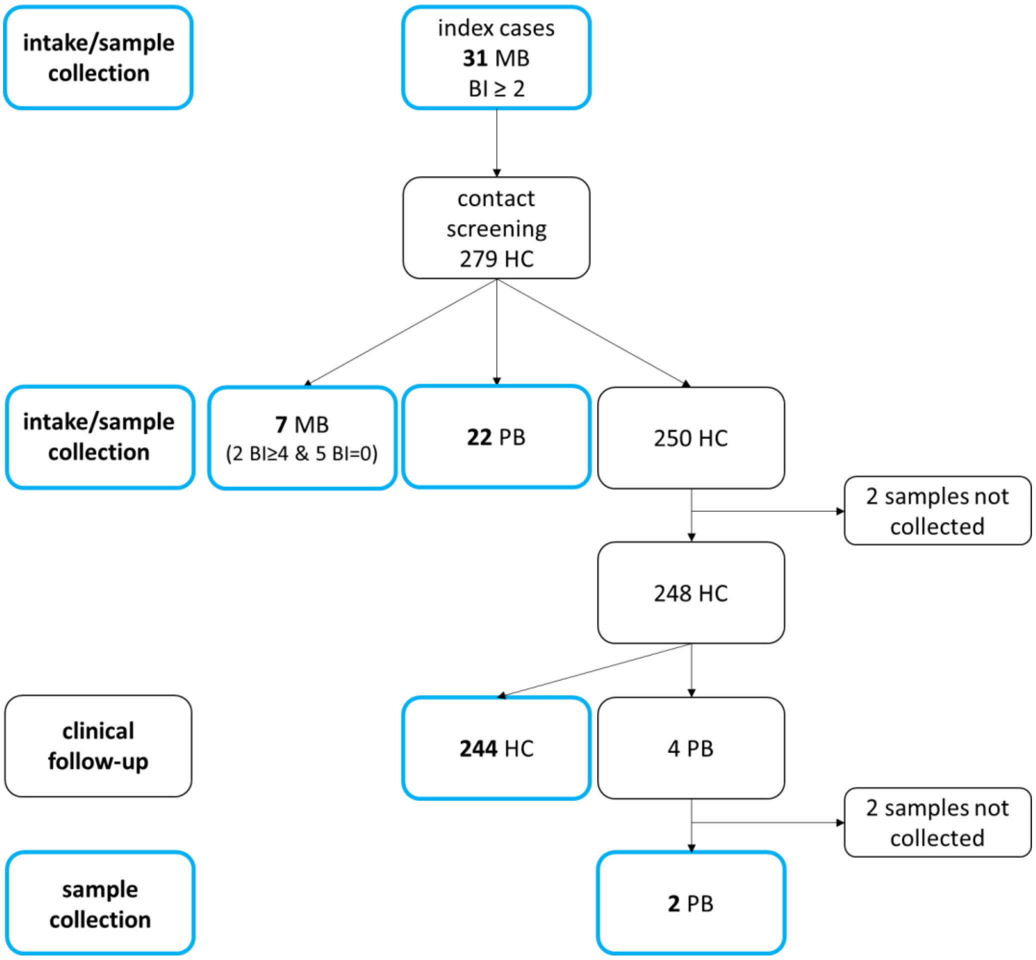

Supplementary Figure S1: ***Schematic representation of sample collection.*** First, index cases diagnosed with multibacillary (MB) leprosy and a bacteriological index (BI) ≥ 2 were recruited. 279 household contacts (HC) of these index cases were screened for the signs and symptoms of leprosy, of which 7 were diagnosed with MB leprosy and 22 with paucibacillary (PB) leprosy at intake. Subjects included in the study were followed up for surveillance of new case occurrence for ≥ 24 months after sample collection (clinical follow-up), identifying 4 additional PB patients. At clinical follow-up samples of HC that developed leprosy were collected. The blue rectangles indicate the amount of MB (n=38), PB (n=24) and HC (n=244) samples used in this study.

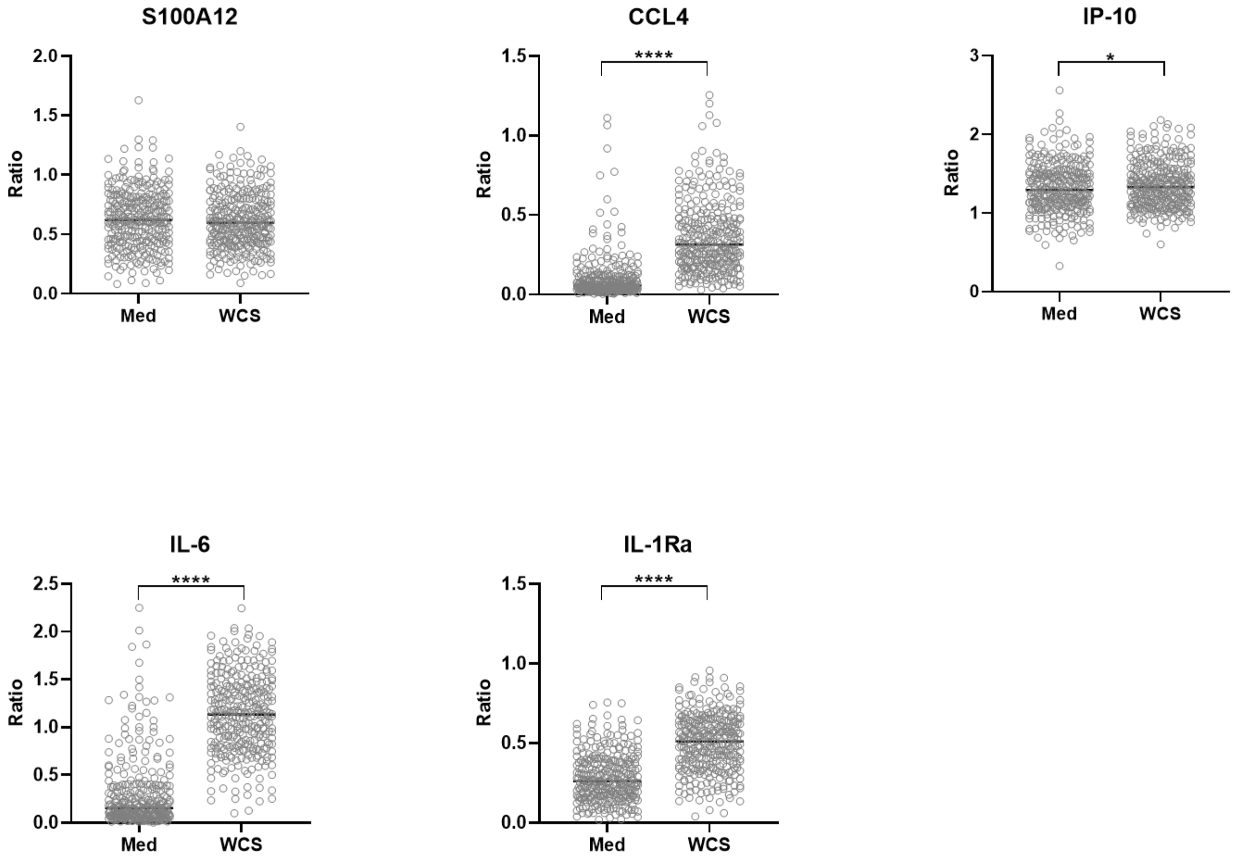

Supplementary Figure S2: ***Influence of whole blood stimulation with Mycobacterium leprae whole cell sonicate (WCS) on biomarker levels.*** S100A12, CCL4, IP-10, IL-6 and IL-1Ra levels were detected in both unstimulated (Med) and *M. leprae* WCS-stimulated (WCS) whole blood assays (WBA) using up-converting phosphor lateral flow assays. Paired comparison between Med and WCS ratio values (*y*-axis; signal detected at the test line divided by the signal at the flow control line) of all study subjects was performed using the Wilcoxon matched-pairs signed rank test. P-values: *$p \leq 0.05$, **$p \leq 0.01$, ***$p \leq 0.001$, ****$p \leq 0.0001$.

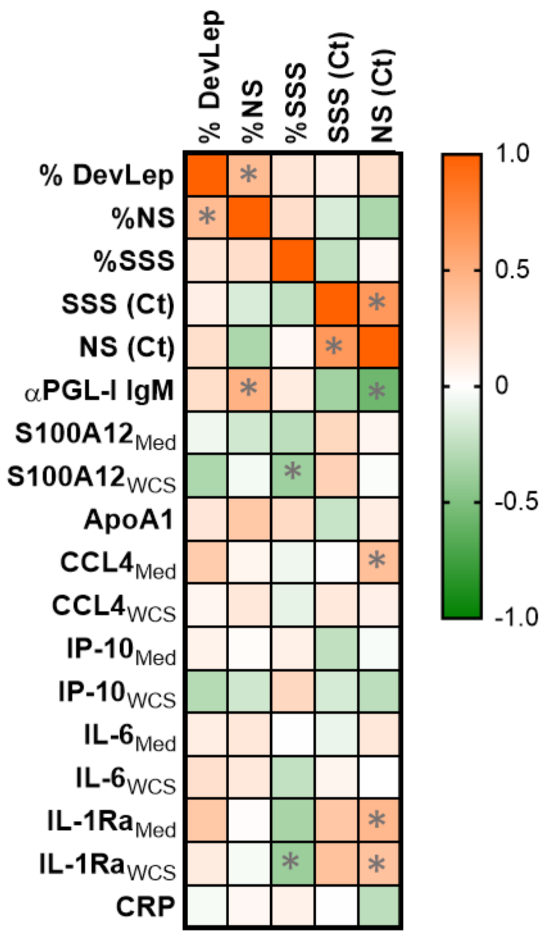

Supplementary Figure S3: ***Correlation of index case characteristics with the development of leprosy and M. leprae colonisation or infection in the same household*****.**

Levels of eight markers in 24 hour *M. leprae* antigen-stimulated whole blood assays (medium = Med, *M. leprae* whole cell sonicate = WCS) were determined for 31 index cases with multibacillary (MB) leprosy (bacteriological index ≥ 2). Per household, the percentage of contacts (HCs) diagnosed with leprosy upon first clinical screening (%DevLep) or with *M. leprae* DNA positivity in nasal swabs (%NS) or skin slit smears (%SSS) was calculated. These percentages and the RLEP Ct values determined by qPCR in NS and SSS were correlated with the levels of innate immunity markers. The heatmap indicates the correlation coefficient (R), ranging from -1 (green) to 1 (orange). Significant correlations ($p < 0.05$) are indicated with a grey asterisk (*), highly significant correlations ($p<0.0001$) are indicated with a black asterisk (*).

References

Tio-Coma, M., Avanzi, C., Verhard, E.M., Pierneef, L., van Hooij, A., Benjak, A., et al. (2020). Detection of new *Mycobacterium leprae* subtype in Bangladesh by genomic characterization to explore transmission patterns. 2020.2003.2005.20031450. doi: 10.1101/2020.03.05.20031450 %J medRxiv.
